# Towards new care strategies in a suburban healthcare center in La Matanza, Argentina: performance evaluation of rapid diagnostic tests for Chagas disease in a non-endemic area

**DOI:** 10.3389/fcimb.2026.1822255

**Published:** 2026-05-19

**Authors:** Margarita Maria Catalina Bisio, Rocío Rivero, María Clara Corso, Analía Gabriela Níttolo, Andrea Ferreira, Ainoha Vilariño, Adriana Calbosa, Sofía Belén Drago, Ulises Rebelo, Yanina Rabi, Alberto Marani, Rita Soledad Digirolamo, Juan Miguel Burgos, Gabriela Vanesa Levy

**Affiliations:** 1 Instituto Nacional de Parasitología “Dr. Mario Fatala Chaben”, Administración Nacional de Laboratorios e Institutos de Salud “Dr. Carlos G. Malbrán”, Buenos Aires, Argentina; 2 Consejo Nacional de Investigaciones Científicas y Técnicas (CONICET), Buenos Aires, Argentina; 3 Instituto de Estudios para el Desarrollo Productivo y la Innovación, Universidad Nacional de José Clemente Paz, José C. Paz, Argentina; 4 Instituto de Investigaciones Biotecnológicas, Universidad Nacional de San Martín (UNSAM) - Consejo Nacional de Investigaciones Científicas y Técnicas (CONICET), San Martín, Argentina; 5 Escuela de Bio y Nanotecnologías (EByN), Universidad Nacional de San Martín, San Martín, Argentina; 6 Comisión de Investigaciones Científicas de la Provincia de Buenos Aires (CIC), La Plata, Argentina; 7 Universidad Nacional de La Matanza, Departamento de Ciencias de la Salud, San Justo, Argentina; 8 Hospital Interzonal General de Agudos Dr. Diego Paroissien, Isidro Casanova, Buenos Aires, Argentina

**Keywords:** Chagas disease, diagnosis, discrete typing unit (DTU), heart disease, parasitemia, persistent infection, rapid diagnostic tests (RDT), *Trypanosoma cruzi (T. cruzi)*

## Abstract

**Introduction:**

Chagas disease, caused by the protozoan parasite *Trypanosoma cruzi*, remains a major public health problem, affecting approximately seven million people worldwide. Although historically restricted to endemic regions, migration has led to its global distribution, increasing the relevance of congenital transmission in non-endemic areas. If not timely diagnosed and treated, infection can progress to chronic Chagas cardiomyopathy in nearly 30 % of patients, generating substantial social and healthcare burdens. Diagnosis requires concordant results from two or three serological assays based on different antigenic principles, limiting timely access to care in peripheral settings.

**Methods:**

We aimed to characterize the clinical, parasitological, and sociodemographic profile of individuals with *T. cruzi* infection in La Matanza (Buenos Aires Province, Argentina), and to assess the diagnostic performance of two rapid diagnostic tests (RDTs) from serum samples in this at-risk population.

**Results:**

In this cross-sectional prospective study including 103 subjects, epidemiological analysis revealed that most infected individuals originated from endemic areas, with a substantial proportion of infections consistent with congenital transmission. Clinical evaluation identified electrocardiographic abnormalities in 30.9 % of infected patients, predominantly right bundle branch block. Molecular characterization showed parasites belonging to DTU V (Discrete Typing Unit V) in 7 infections (one coinfected with DTU VI). RDT1 showed a sensitivity of 82.5 % and specificity of 100 %, whereas RDT2 exhibited a sensitivity of 87.5 % and specificity of 85.7 %.

**Discussion:**

Overall, our findings provide a comprehensive characterization of *T. cruzi* infected individuals in a non-endemic region with ongoing transmission dynamics shaped by migration and congenital infection, pointing out advantages and limitations of RDT use in peripheral healthcare settings.

## 1 Introduction

Chagas Disease (CD), caused by the parasitic protozoan *Trypanosoma cruzi*, is a major public health concern in Latin America. The infection is endemic in 22 American countries (Beatty et al., 2025), has a prevalence of 7 million people, an incidence of 40,000 (World Health Organization, 2023), and puts 100 million at risk of infection (Echeverría et al., 2020). Moreover, around 100,000 infected people live outside endemic countries, mainly in Europe, where more than 4.6 million migrants from endemic countries live regularly (Gonzalez-Sanz et al., 2023). Vector-borne is the main route of infection in rural endemic areas. However, congenital transmission has become particularly significant due to population movements that introduced infection to urban centers beyond endemic regions, constituting a new problem in migration-receiving cities and countries (Schmunis and Yadon, 2010).

*Trypanosoma cruzi* infection evolves from an acute phase, characterized by high parasitemia and usually non-specific symptoms, to an asymptomatic chronic phase distinguished by low parasitemia and presence of IgG antibodies, which can last for years and even the entire life of the patient. Over the years, 30–40% of these patients progress to CD, characterized by cardiac and/or gastrointestinal manifestations (Pérez-Molina and Molina, 2018). Evidence of heart disorder progression usually involves electrocardiographic (ECG) abnormalities mainly right bundle branch block, left anterior fascicular block, and diffuse ST-T changes characteristic of chronic Chagas cardiomyopathy (Ribeiro et al., 2013). Progression to the cardiac form of the disease may be due to several host and parasite characteristics (Pérez-Molina and Molina, 2018).

In 2009, based on genetic variability, seven different groups were defined within *T. cruzi* including six discrete typing units (DTUs I to VI) and TcBat (Marcili et al., 2009; Zingales et al., 2009; Brenière et al., 2016). Several studies have shown that these groups have dissimilar virulence, antigenicity, and geographical distribution. Related to human infection, several DTUs have been identified in different biological samples (Burgos et al., 2007; Messenger et al., 2015; Brenière et al., 2016; Villanueva-Lizama et al., 2019).

Diagnosis in the acute and congenital phases of infection is established by direct visualization of parasites in the blood or after PCR amplification. On the other hand, in the chronic phase, serological tests such as ELISA, indirect hemagglutination (IHA) or indirect immunofluorescence (IIF) are used for detecting IgG antibodies against *T. cruzi* (Lopez-Albizu et al., 2023; Schijman et al., 2024). According to the Pan American Health Organization’s (PAHO) guidelines for the diagnosis and treatment of CD, *T. cruzi* infection must be confirmed using the diagnostic gold standard defined as reactive results in at least two conventional assays based on different principles and antigens. Accepted combinations include ELISA–IHA, ELISA–IIF, or IHA–IIF. These tests must be conducted in parallel, and if only one assay yields a reactive result, a third test not used in the initial evaluation should be performed (PAHO, 2019).

Nevertheless, the implementation of these diagnostic tests remains beyond the capacity of many healthcare facilities and, frequently, biological samples must be referred to specialized laboratories equipped with appropriate technology and staffed by qualified personnel (Klein et al., 2017). Moreover, patients are typically required to attend the healthcare facilities, often far from the place of residence, on at least two occasions (for blood sample collection and subsequently for the retrieval of results) which not only increases the overall cost of diagnosis but also heightens the risk of patient attrition or loss (Klein et al., 2017; Pinazo et al., 2021). This complex care landscape contributes to substantial leads to an underdiagnosis currently estimated at only 10%, resulting in only 1% of patients being properly treated (Sanmartino et al., 2015; PAHO, 2022). This scenario prompted the World and Health Organization (WHO) to declare the need to “simplify and update diagnostic algorithms to improve access and shorten diagnosis time”, highlighting the need to develop diagnostic systems appropriate for primary care centers (World Health Organization, 2020).

In recent decades, immunochromatography tests have emerged as rapid diagnostic tests (RDTs) due to their advantages. Among them, the dispensability of qualified personnel, sophisticated equipment and refrigeration of the reagents and, especially, the virtue of the speed of obtaining the result and the simplicity and cost-effectiveness of their use (Sánchez-Camargo et al., 2014; Pinazo et al., 2021). According to PAHO guidelines, RDTs should be restricted to screening purposes, and reactive results must be confirmed using conventional serological assays (PAHO, 2019). Lastly, with improvements achieved in RDTs, the scientific community has been evaluating the possibility of incorporating RDTs into a diagnostic algorithm to facilitate it, especially in low-complexity health centers. Recently, some studies have evaluated RDTs in different populations and samples, with the aim of using them as point-of-care testing (POCT) (Sánchez-Camargo et al., 2014; Lozano et al., 2019; Marchiol et al., 2023; Rivero et al., 2024). Expert consensus has emphasized that performance of RDTs must be geographically validated, with evaluation criteria defined at the country or subregional level, and adapted to local bioecogeographic contexts, *T. cruzi* genetic diversity (DTUs), and the characteristics of the populations undergoing screening (Perez et al., 2024).

In Argentina, according to the National Ministry of Health, there are between one and three million infected individuals, 30% of whom present different degrees of cardiac alterations (Ministry of Health of Argentina, 2018; PAHO, 2019). Related to the Metropolitan Area of Buenos Aires (MABA), a non-endemic region for *T. cruzi* infection, few reports describing the epidemiological situation of CD are available (Stagnaro et al., 2014). A recent retrospective characterization of a patient cohort from La Matanza district describes the complexity of healthcare delivery in socioeconomically disadvantaged communities, the difficulty of completing diagnosis and carrying out parasiticidal treatment with follow-up, as well as the scarcity of epidemiological and registered data in the region (Vilariño et al., 2025). La Matanza, with an area of ​​329 km² and 1.84 million inhabitants, is the most populated district in Buenos Aires Province. Of these, nearly half a million are migrants from *T. cruzi* endemic regions, with more than 300,000 coming from other Argentine provinces and more than 150,000 from other endemic countries (39% from Paraguay, 16% from Bolivia, 8% from Peru, 5% from Venezuela, among others). Although no data have been reported on *T. cruzi* infection or CD prevalence among La Matanza residents, population statistics suggest a high rate, which also includes nearly half a million women of childbearing age, a subpopulation that should be specifically evaluated to prevent and/or treat congenital transmission (Instituto Nacional de Estadística y Censos (INDEC), 2022).

Considering this background, we aimed to characterize the profile of individuals affected by *T. cruzi* infection in La Matanza who attended the Hospital Diego Paroissien, by evaluating serological status, parasitemia, infecting parasite DTUs, cardiac involvement, and sociodemographic characteristics. Additionally, we assessed the diagnostic performance of two RDTs in a population at risk for CD residing in this non-endemic region.

## 2 Materials and methods

### 2.1 Study design and subject selection

**Figure 1:**
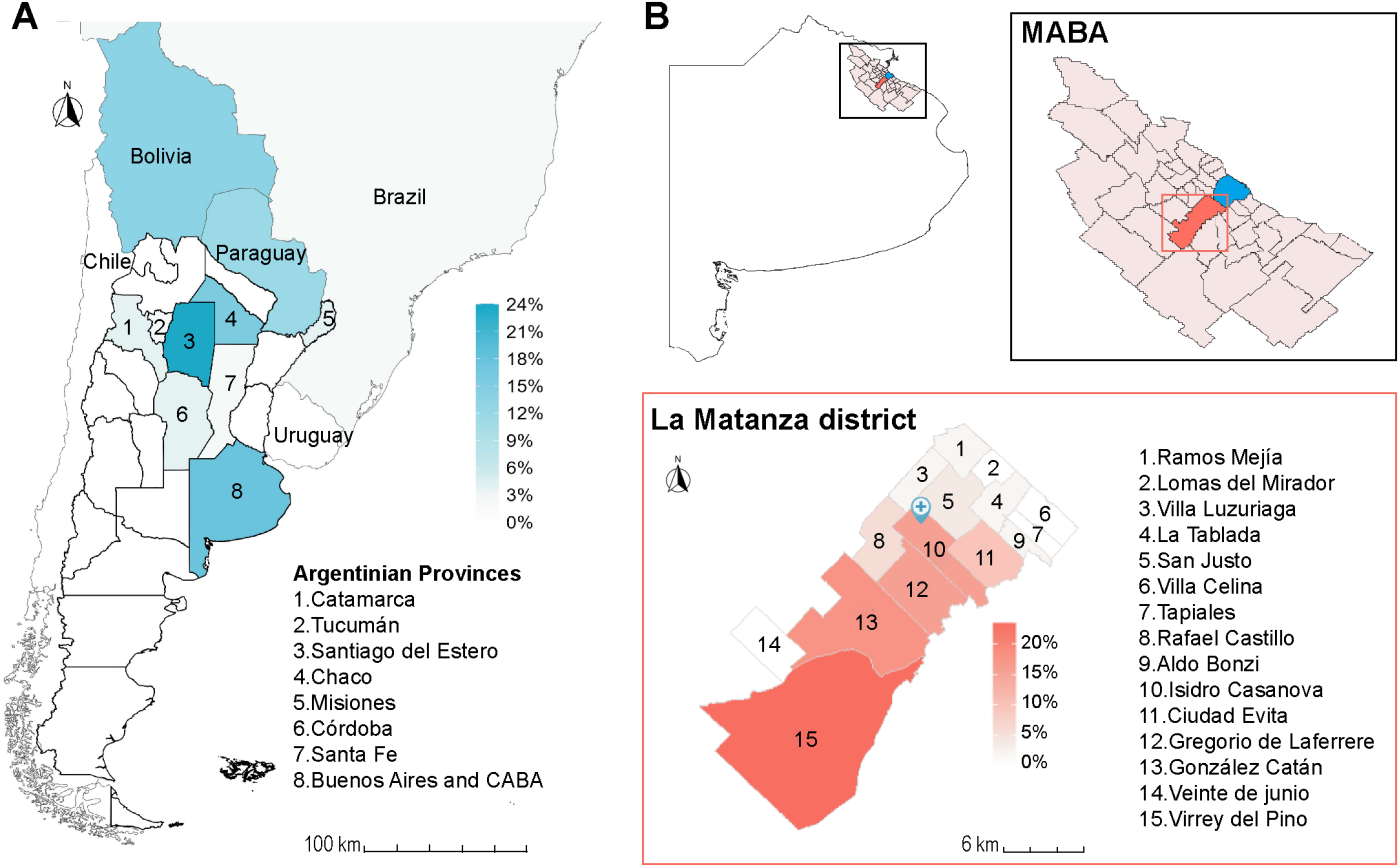
Distribution of seropositive individuals. **(A)** Place of birth; **(B)** upper panel, Buenos Aires Province with the Metropolitan Area (MABA) in the black box; lower panel, locality of residence within La Matanza district (outlined in red, shaded according to the proportion of infected individuals residing in each locality). Hospital Diego Paroissien is located in Isidro Casanova (blue pin).

A cross-sectional prospective study was conducted from June 2023 to December 2024 at the Hospital Diego Paroissien, a tertiary care health center located in La Matanza, in the suburbs of Buenos Aires province, Argentina (**Figure 1**).

**Figure 2:**
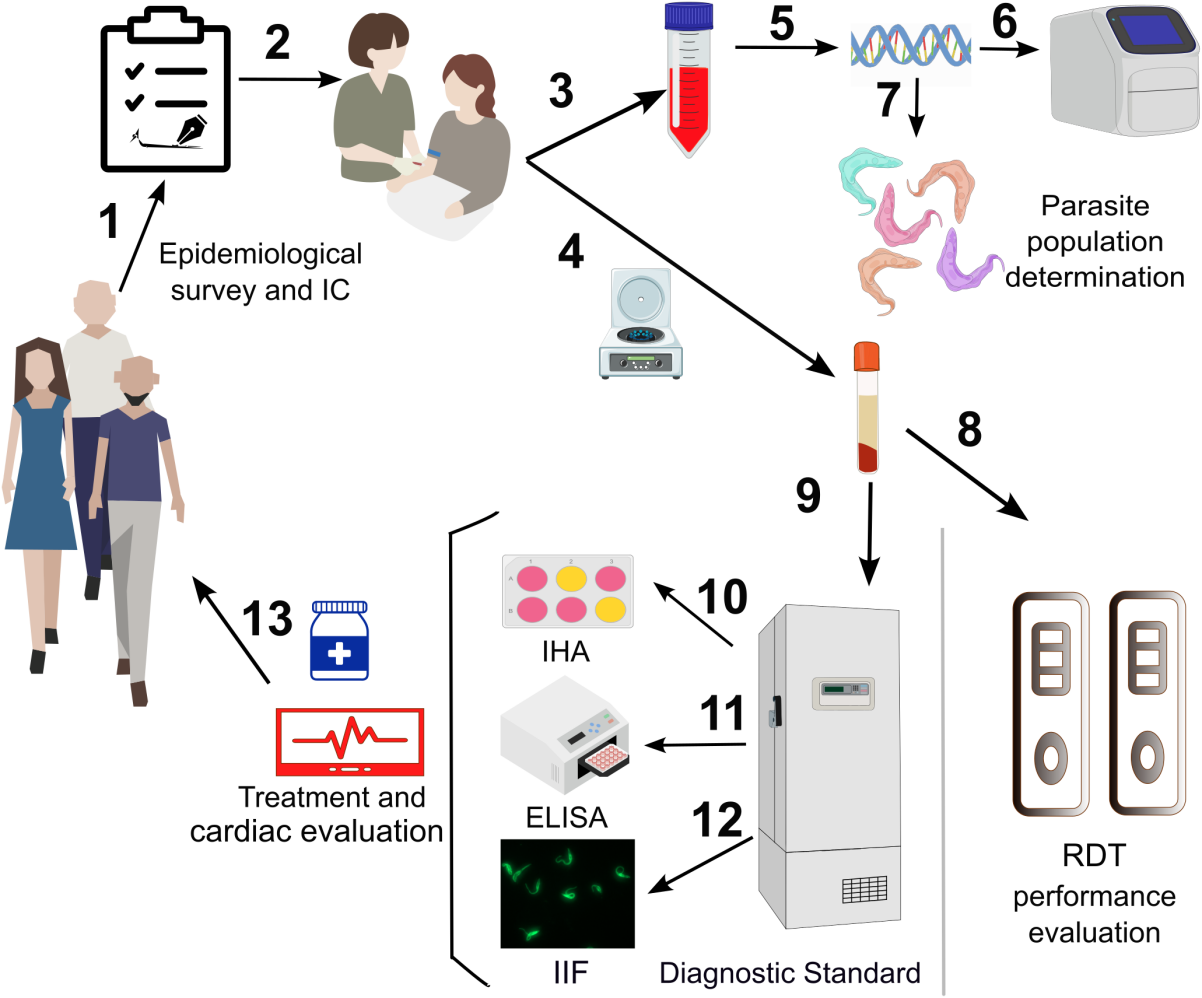
Study design scheme. Invited participants underwent a clinical and epidemiological survey and signed informed consent (IC) [1]. Peripheral blood was collected [2] and mixed with guanidine buffer [3] for DNA extraction [5] to measure parasitemia levels by qPCR [6] and Discrete Typing Unit (DTU) determination by end-point PCRs [7]. In parallel serum was obtained [4] for Rapid Diagnostic Test (RDT) evaluation [8] and stored [9] until diagnostic standard tests were performed (IHA [10] and ELISA [11], and IIF [12] if required). Patients with a positive diagnosis underwent clinical and cardiological evaluation and were referred for assessment of etiological treatment eligibility [13]. This figure was created using original illustrations and elements licensed under the Creative Commons Attribution 4.0 International License (CC BY 4.0; https://creativecommons.org/licenses/by/4.0/), and includes adapted material from NIAID Visual & Medical Arts, NIH BioArt Source (https://bioart.niaid.nih.gov/bioart/634), as well as icons from UXWing (https://uxwing.com/).

Subjects attending Central Laboratory or Departments of Cardiology or Infectious Diseases with suspected *T. cruzi* infection (i: born or lived in an endemic area; ii: born to a seropositive woman; iii: recipients of blood transfusions or organ transplants) were invited to participate (**Figure 2**). Recruiters used a brief screening questionnaire to assess eligibility for enrollment in the study and to collect clinic and epidemiological data. Patients with laboratory-confirmed diagnoses were contacted by telephone and assessed by the Department of Infectious Diseases for treatment eligibility, and underwent cardiological evaluation, including ECG and Doppler echocardiography in the Department of Cardiology.

Furthermore, for RDTs performance evaluation, the inclusion criteria were age between 18 and 75 years old and be willing to donate a serum sample for RDTs. The exclusion criteria were previous treatment with trypanocidal drugs for CD or the presence of immunosuppression.

### 2.2 Ethical considerations

The study was approved by the Municipal Commission of Bioethics of the Municipality of La Matanza (COMUBI) and registered with the Provincial Directorate of Hospitals of Buenos Aires Province (CEI N° 056). Patients who wished to participate provided written informed consent. The study was conducted according to the national standards in compliance with the current good clinical practice guidelines.

As primary epidemiological data (origin, age, gender, possible route of infection, etc.) and personal information were collected from the participants, their identities were coded. All RDT devices and samples were coded and identified prior to use.

### 2.3 Data collection

Epidemiological, clinical and demographic data were collected at enrollment using a standardized questionnaire. The information included the date and place of birth, current place of residence, and maternal place of birth. Data related to potential risk factors for *T. cruzi* infection were also recorded, including residence or prolonged stay in endemic areas, and previous blood transfusions.

### 2.4 Samples

A volume of 3 to 8 mL of peripheral blood was collected at the time of enrollment. When 8 mL were obtained, 5 mL were immediately mixed with an equal volume of GEB buffer (6 M Guanidine-Hydrochloride/0.2 M EDTA) and stored at room temperature until DNA extraction (Avila et al., 1991) for parasitemia and DTU determinations. The remaining 3 mL of blood was centrifuged for 15 min at 3000 rpm to obtain the serum fraction, which was stored at -20 °C until reference standard serology and RDTs were performed (**Figure 2**).

### 2.5 Reference standard

At time of recruitment, serology for *T. cruzi* was performed at the Hospital Diego Paroissien according to recommendations of the national guidelines for patient care consisting of two standardized tests in parallel, mainly hemagglutination inhibition assays (IHA Chagas Polychaco^®^) and enzyme-linked immunosorbent assays (ELISA, Chagatest lisado, Wiener Lab^®^). A third test in case of discordance (indirect immunofluorescence (IIF, Inmunofluor Chagas Bioscientifica^®^) was performed at the National Institute of Parasitology (Ministry of Health of Argentina, 2018). Subjects were considered infected when two serological tests showed concordant reactive results in serological assays, whereas concordant non-reactive results in two tests were considered indicative of the absence of infection (**Figure 2**).

### 2.6 Index tests

The WL Check Chagas (WL) Wiener Laboratorios^®^ (Argentina) and the Chagas Detect™ *Plus* Rapid Test (CDP) InBios International^®^ (USA) immunochromatographic RDTs were performed. For WL, 40 µL of serum samples were added, followed by the addition of 3 drops of diluent, with results read after 35 min. For CDP, 5 µL of serum were used, followed by the addition of 1 drop of gold solution, incubation for 5 min, subsequent addition of 1 drop of Chase Buffer Type A, and result interpretation after 15 min. Positive or negative results were defined by the presence or absence of specific bands in the chromatography, respectively. Invalid result was defined as the absence of a visible control line. Laboratory technicians ascertaining the RDTs results were blinded to the results of the reference standard.

### 2.7 Parasitemia and parasite population determinations

For molecular studies, DNA was isolated from GEB blood samples using the High Pure PCR template preparation kit (Roche) following manufacturer´s instructions, with the elution step performed with 100 μL of elution buffer (Moreira et al., 2013). A multiplex real-time PCR (qPCR) assay capable to detect the *T. cruzi* nuclear satellite and internal positive control DNA in a single-tube reaction previously analytically validated was performed (Bisio et al., 2021) (**Figure 2**). Briefly, the qPCR reactions were carried out with 5 μL of eluted DNA, using FastStart Universal Probe Master Mix (Roche Diagnostics GmbHCorp, Mannheim, Germany) in a final volume of 20 μL. The amplifications were performed using 750 nM of the cruzi1 and cruzi2 primers, 50 nM of the cruzi3 probe, 100 nM of the IACTqFw and IACTqRv primers, and 50 nM of the IACTq probe. The TaqMan MGB cruzi3 and IACTq probes were labeled with FAM and VIC dye, respectively. The cycling conditions were as follows: 10 min at 95 °C, and 40 cycles of 15 s at 95 °C, followed by 1 min at 58 °C. The amplifications were carried out in a Bio-Rad CFX96 Real Time PCR System (Bio-Rad, USA).

The PCR-positive samples were subsequently evaluated to determine the infecting parasite DTU (**Figure 2**) by two different end-point PCRs approaches: *i*) Burgos et al. algorithm classification based on Splice Leader, 24Sα-rDNA and A10 fragment PCR amplifications (Burgos et al., 2010); *ii*) Bontempi et al. strategy based on nested-specific kDNA amplifications (Bontempi et al., 2016).

### 2.8 Data analysis

Primary epidemiological, demographic and clinical data were collected using surveys. Maps were generated using QGIS (QGIS Geographic Information System, Open Source Geospatial Foundation; http://qgis.org) from vector layers provided by Natural Earth, the Instituto Geográfico Nacional and GeoARBA (Infraestructura de Datos Espaciales). https://www.naturalearthdata.com, https://www.ign.gob.ar/ and geo@arba.gov.ar.

The data collected and serological results (index test and reference standard) were registered in an Excel database. ELISA results were expressed as a positivity index (PI), calculated as the ratio between the optical density (OD) obtained for each individual sample and the assay-specific cutoff value (OD_sample/OD_cutoff), according to the manufacturer’s instructions. Samples were classified as reactive when the positivity index was ≥1.0 and non-reactive when <1.0. To evaluate the performance of RDT (diagnostic accuracy), sensitivity (Se) and specificity (Sp) were calculated using the following formulas: Se= True Positive/(True Positive + False Negative), Sp= True Negative/(True Negative + False Positive). Differences in ELISA titers expressed as PI between RDT-positive and RDT-negative samples were additionally compared using the Mann–Whitney U test.

## 3 Results

### 3.1 Characteristics of the studied population

A total of 103 patients were enrolled in this prospective study performed in La Matanza, a non-endemic region for CD. The studied population (68 women/35 men) had a mean age of 47.1 years (range 18-83). Among them, 52 patients were infected with *T. cruzi* (50.5%) as determined by positive results in reference tests (ELISA and IHA, and IIF in case of discordant results) (**Table 1**). Of these, 7 (13.5%) were born in Bolivia, 6 (11.5%) in Paraguay, 1 (1.9%) in Brazil and 38 (73.1%) in Argentina, mainly in the provinces of Santiago del Estero (12, 23.1%) and Chaco (9, 17.3%) and in the MABA (9, 17.3%), including the Autonomous City of Buenos Aires (CABA) and its suburbs (**Figure 1A**). The regions of Argentina or countries from which the participants migrated, as well as the risk factors that prompted CD testing, are detailed in **Table 1**. Patients came to the hospital from different localities in La Matanza (mostly from Virrey del Pino, Gregorio de Laferrere, Gonzalez Catán and Isidro Casanova localities, **Figure 1B**), some of which are located up to 20 km away from the health center. Indeed, the largest number of infected patients resided in the most geographically remote areas served by the hospital (**Figure 1B**).

**Table 1:** Characteristics of the studied population.

Variable	Total	*T. cruzi* negative	*T. cruzi* positive
	N=103	N=51	N=52
	N (%)	N (%)	N (%)
Median age (IQR)	47.1 (35.0-59.0)	41.6 (29.0-51.5)	52.4 (46.0-60.0)
Biological sex			
Female	68 (66.0)	35 (68.6)	33 (63.5)
Male	35 (34.0)	16 (31.4)	19 (36.5)
Migration			
Country or region of Birth			
Argentina	79 (76.7)*	41 (80.4)	38 (73.1)*
Metropolitan Area of Buenos Aires	45 (57.7)	36 (87.8)	9 (24.3)
Northwestern region	19 (24.4)	4 (9.8)	15 (40.6)
Northeastern region	12 (15.4)	1 (2.4)	11 (29.7)
Pampas region	2 (2.5)	0 (0)	2 (5.4)
Bolivia	13 (12.6)	6 (11.8)	7 (13.5)
Paraguay	7 (6.8)	1 (1.9)	6 (11.5)
Brazil	1 (1.0)	0 (0)	1 (1.9)
NA	3 (2.9)	3 (5.9)	0 (0)
Transfusion			
No	72 (69.9)	37 (72.6)	35 (67.3)
Yes	22 (21.4)	8 (15.7)	14 (26.9)
NA	9 (8.7)	6 (11.7)	3 (5.8)
Mother born in an endemic area			
Yes	84 (81.6)	35 (68.6)	49 (94.2)
No	12 (11.6)	12 (23.5)	0 (0)
NA	7 (6.8)	4 (7.9)	3 (5.8)

IQR, interquartile range; NA, Not available; Metropolitan Area of Buenos Aires, Autonomous City of Buenos Aires (CABA) and suburban districts; Northwestern region, Catamarca, Santiago del Estero and Tucumán provinces; Northeastern region, Chaco, Corrientes and Misiones provinces; Pampas region, Córdoba and Santa Fe provinces.

*Province of origin was unknown for one Argentine patient.

### 3.2 Cardiac manifestations

Out of 52 seropositive patients, 42 (80.8%) underwent cardiological evaluation. Among them, electrocardiographic abnormalities were detected, exclusively or in combination, in 13 patients (30.9%). The main observed cardiomyopathies were right bundle branch block (RBBB, 69.2%), premature atrial contraction (PAC, 46.1%) and premature ventricular contraction (PVC, 38.5%). supraventricular tachycardia (SVT), non-sustained ventricular tachycardia (NSVT) and atrial fibrillation (AF) were also observed. The number of patients presenting these and other less frequent electrocardiographic abnormalities is detailed in **Table 2**. On the other hand, dilated cardiomyopathy was observed in 4 out of 34 (11.7%) patients who underwent Doppler echocardiographic evaluation. Of these, two presented some of the aforementioned electrocardiographic alterations (not shown).

**Table 2:** Electrocardiographic abnormalities in patients with chronic *T. cruzi* infection.

RBBB	PAC	PVC	SVT	NSVT	AF	AV block	TBS	N	Gender (M/F)
+								4	2/2
+	+	+						2	1/1
+	+					+		1	1/0
+	+	+	+					1	1/0
+					+			1	1/0
		+		+				1	0/1
		+		+	+			1	1/0
	+		+					1	0/1
	+		+	+			+	1	0/1
**69.2%**	**46.1%**	**38.5%**	**23.1%**	**23.1%**	**15.4%**	**7.7%**	**7.7%**		

RBBB, Right Bundle Branch Block; PAC, Premature Atrial Contraction; PVC, Premature Ventricular Contraction; SVT, Supraventricular Tachycardia; NSVT, Non-Sustained Ventricular Tachycardia; AF, Atrial Fibrillation; AV Block, Atrioventricular block; TBS, Tachy-Brady Syndrome; N, number of patients with single or combined cardiomyopathies. M, male; F, female. Lower row (bold), frequency of cardiac abnormalities.

### 3.3 Parasitological findings: parasitemia and *T. cruzi* DTUs

Parasitemia was assessed by qPCR in 60 GEB blood samples (13/51 non-infected subjects and 47/52 infected). All samples from non-infected individuals were negative for qPCR (13/13; 100%, 95% CI: 77.2-100). Among infected patients, 11/11 (100%, 95% CI: 74.1-100) previously treated with antiparasitic drugs also yielded negative results, whereas 14/36 (38.9%, 95% CI: 18.0-46.9) of untreated infected individuals had positive qPCR findings.

For *T. cruzi* characterization, all DNA samples with positive qPCR results were evaluated for DTU identification. Due to the lower sensitivity of typing reactions, two endpoint PCR strategies were performed to rescue the largest possible number of positive findings, detecting 6 patients with Tc V infections and 1 with a mixed Tc V+VI infection.

### 3.4 Diagnostic performance of RDTs

**Figure 3:**
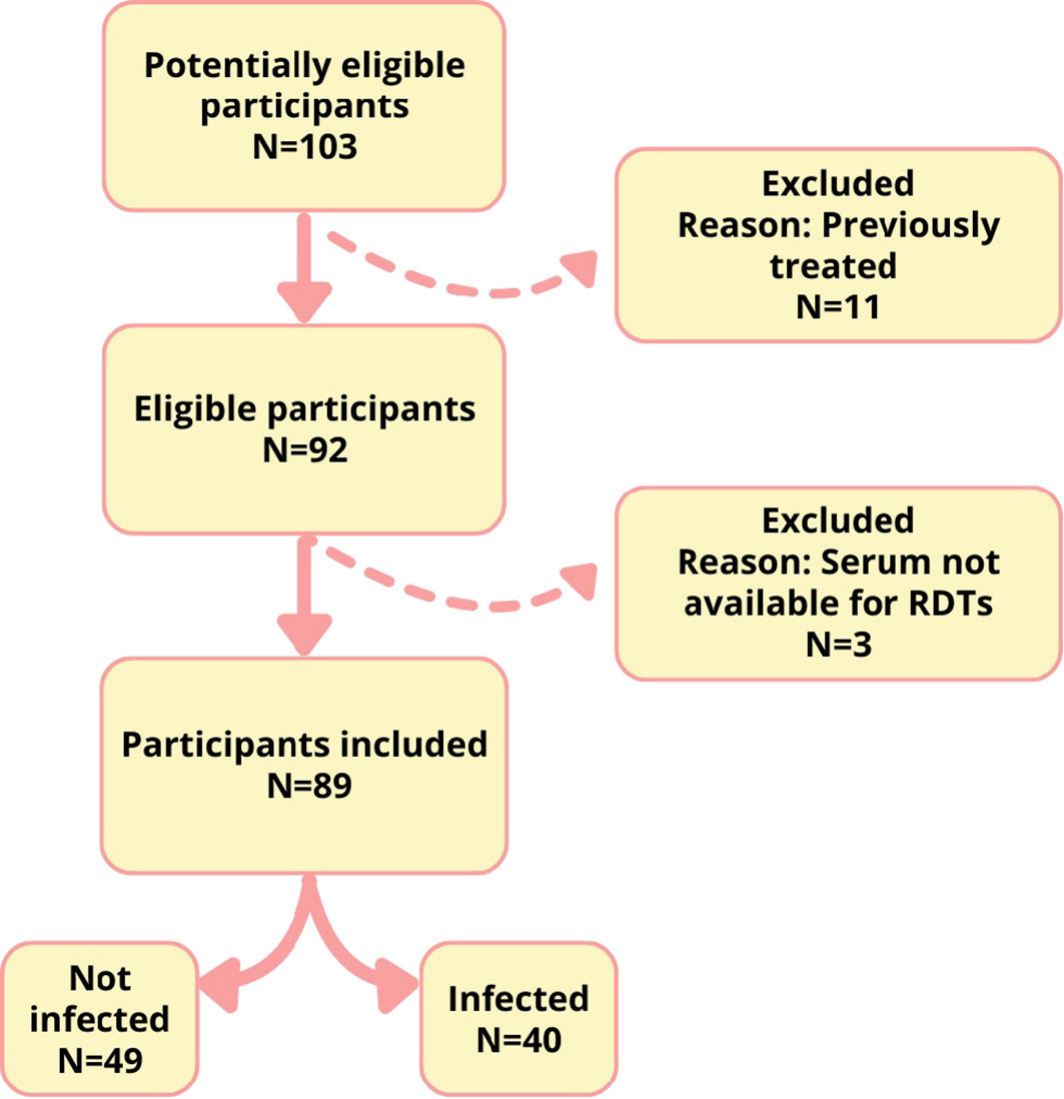
Flow diagram for RDTs performance evaluation.

Among 103 studied subjects, 11 were excluded for RDT performance evaluation because they reported previous treatment for CD, and another 3 were excluded because serum samples were unavailable to perform analysis. Among 89 included patients, 40 presented *T. cruzi* seropositive findings and 49 were not infected (**Figure 3**). A total of 7/89 (7.9%) individuals exhibited discordant ELISA and IHA results, requiring IIF for diagnostic resolution. Of these discordant cases, 6/7 were IIF-positive and 1/7 was IIF-negative.

The performances of both evaluated RDTs (WL and CDP) are depicted in **Table 3**. The WL RDT demonstrated higher specificity (100%, 95% CI: 92.8-100), accuracy (92.1%, 95% CI: 84.4-96.8), and agreement (kappa index: 0.838), whereas the CDP RDT showed slightly higher sensitivity (87.5%, 95% CI: 73.2-95.8). No invalid tests were reported in any RDTs.

**Table 3:** Estimated performance for RDTs.

	N	TP	FN	TN	FP	Se (95% CI)	Sp (95% CI)	Accuracy (95% CI)	Kappa index (95% CI)
WL	89	33	7	49	0	82.5% (67.2-92.7)	100% (92.8-100)	92.1% (84.4-96.8)	0.838 (0.725-0.952)
CDP	89	35	5	42	7	87.5% (73.2-95.8)	85.7% (72.8-94.1)	86.5% (77.7-92.8)	0.729 (0.586-0.871)

WL, WL Check Chagas, Wiener Laboratorios®; CDP, Chagas Detect™ Plus Rapid Test, InBios International; TP, true positive; FN, false negative; TN, true negative; FP, false positive; Se, sensitivity; Sp, specificity; 95% CI, 95% confidence interval.

Notably, samples yielding false-negative results with the WL index test (N = 7, **Table 3**) showed significantly lower ELISA positivity index (PI), with a median of 2.25 (IQR: 1.21–3.28), compared with true-positive samples, which had a median of 9.68 (IQR: 6.79–11.33; p=0.0015). Similarly, samples with false-negative results using the CDP index test (N = 5, **Table 3**) demonstrated lower ELISA reactivity (median PI: 1.35; IQR: 1.06–2.25) than true-positive samples (median PI: 9.68; IQR: 6.60–11.24; p=0.0006). Regarding false-positive results observed with the CDP RDT (N = 7, **Table 3**), 6/7 samples had been non-reactive by both ELISA and IHA, whereas 1/7 exhibited discordant serology (ELISA-positive, IHA-negative) with a negative IIF result.

## 4 Discussion

Diagnosis of chronic *T. cruzi* infection requires concordant results from two serological assays based on different antigenic principles, and occasionally a third test to resolve discordant findings. It is therefore limited to centralized healthcare settings. This algorithm delays diagnosis, prolongs turnaround times, and increases the risk of missed opportunities for timely access to care and treatment. In absence of early etiological treatment, chronic *T. cruzi* infection remains asymptomatic in approximately 70% of cases. The remaining 30% develop clinical manifestations within 10–30 years, with Chagas cardiomyopathy being the most prevalent and severe manifestation (Echeverría et al., 2020).

The presence of CD in large urban centers has been poorly characterized in Argentina. Although 36% of the population resides in the MABA (Instituto Nacional de Estadística y Censos (INDEC), 2022), only a few studies have addressed the characteristics of *T. cruzi* infection in this so-called “conurbano” marked by high population density and pronounced socioeconomic heterogeneity. Retrospective studies conducted in this region have shown delayed diagnoses and limited population awareness of the long-term health consequences of the disease (Stagnaro et al., 2014; Vilariño et al., 2025). This prospective cross-sectional study focused on CD at a tertiary healthcare center in La Matanza, the most populous district in MABA characterized by a high proportion of migrants from endemic regions. Indeed, 80.8% of infected individuals were migrants from other Argentine provinces or neighboring countries (**Table 1**, **Figure 1A**). Noteworthy, 9 infected patients (17.3%) were born in MABA. Interestingly, 7 of them had mothers who were born in endemic areas or reported travel to endemic regions, which strongly suggests congenital transmission.

Clinical trials have demonstrated high rates of parasitological cure in congenitally infected newborns and in children with early chronic *T. cruzi* infection, leading to changes in treatment recommendations in several countries (Villar et al., 2014; Altcheh et al., 2021). In addition, the importance of treating women of reproductive age to prevent mother-to-child transmission has been emphasized, highlighting the need for systematic screening of pregnant women in childbearing age in addition to early treatment of infected children (Sosa-Estani et al., 2009; Moscatelli et al., 2015). Furthermore, family clustering and second-generation congenital transmission (grandmother–mother–newborn) are well-documented phenomena, particularly in non-endemic areas, underscoring the importance of screening strategies in women of childbearing age, newborns, and siblings of confirmed cases (Sánchez Negrette et al., 2005). Timely detection of infection, ideally during childhood, can prevent long-term complications. In the population studied, diagnosis occurred during chronic infection adulthood, a phase at which the efficacy of available drugs (benznidazole and nifurtimox) is less well established, and evidence from randomized controlled trials remains insufficient to fully support their use (Vallejo et al., 2020). Indeed, regarding patient clinical data, cardiac abnormalities consistent with CD were identified. However, one limitation of this study is that digestive manifestations of the disease were not assessed.

Real-time PCR shows high sensitivity for *T. cruzi* detection in scenarios characterized by high parasitemia, such as congenital, oral, or reactivation infections. In our study, qPCR analysis presented a positivity of 38.9% (14/36), comparable to that reported for a chronic migrant population attending a healthcare center in Spain (42%) (Sulleiro et al., 2020). Parasitemia levels were below the quantification limit (data not shown), corresponding to cycle threshold (Ct) values above the quantification cutoff (5 parasite equivalents/mL) (Bisio et al., 2021) in concordance with parasitemia values reported in Argentina for chronic *T. cruzi* infection (Moreira et al., 2013).

Regarding infecting parasite DTUs, Tc V, the main variant found herein, as well as Tc VI, are frequent populations present in blood samples of individuals from the Southern Cone, where patients included in this study came from. Moreover, these parasite groups have frequently been associated with cardiomyopathy and congenital infections (Messenger et al., 2015). As DTUs were evaluated in 38.9% of samples (qPCR positives), observed parasite variability may represent a subset of the circulating parasite diversity in this area of MABA.

Concerning access to etiological therapy, during the study period 9 infected subjects started etiological treatment after receiving their laboratory results in accordance with the criteria established by the Ministerio de Salud de la Nación (2018) (Ministry of Health of Argentina, 2018). Moreover, 11 additional patients had received antiparasitic therapy prior to enrollment, resulting in 38.5% (20/52) of infected individuals having been treated even at this tertiary healthcare center located far from many patients’ residence (**Figure 1B**). This treatment inclusion rate is markedly higher than the ~1% estimated for the general population (Sanmartino et al., 2015), highlighting the critical role of timely diagnosis in enabling access to treatment.

Rapid diagnostic tests offer rapid results, ease of use, and require only small sample volumes. They do not require cold-chain storage or high technical complexity, making them particularly suitable as POC tests (Schijman et al., 2024). Currently, six RDTs for *T. cruzi* diagnosis are available in Argentina and only one is approved by the Food and Drug Administration (CDP). All are cassette-based immunochromatographic assays requiring only a few drops of blood or serum, with results available in less than 35 minutes (Rivero et al., 2024). Rapid diagnostic tests have been used for screening purposes in Bolivia (Egüez et al., 2017), Colombia (Suescún-Carrero et al., 2021), Paraguay and Argentina (Mendicino et al., 2019), with reported sensitivities and specificities ranging from 97% to 100%. In particular, the WL RDT showed sensitivities of 92.5% (84.4–100) and 93% (88.5–96.1%), and specificities of 97.3% (92.8–100) and 99% (96.4–99.9%) in studies conducted in Autonomous City of Buenos Aires (Lopez-Albizu et al., 2020; Rivero et al., 2024). In contrast, our study reported sensitivities and specificities of 82.5% (67.2–92.7) and 100% (92.8–100.0) for WL, and 87.5% (73.2–95.8) and 85.7% (72.8–94.1) for CDP, respectively. However, comparing the clinical performance of RDTs is challenging due to differences in validation processes, sample types and volumes, and reference standards. The performance evaluation of the RDTs was conducted in a healthcare-center-based study population without a specifically designed sampling strategy; therefore, the use of convenience sampling without prior sample size calculation may have limited the robustness of the diagnostic accuracy estimates. Previous assessments were often conducted with pre-characterized serum samples under controlled laboratory conditions that may not reflect real-world settings (Rivero et al., 2024). Therefore, this study was deliberately conducted in a hospital environment using routine serum clinical samples highlighting the importance of evaluating diagnostic performance under real-world healthcare conditions. Although both RDT inserts indicate that the tests may be used with different specimen types, the application of them at the POC may benefit from the use of finger-prick whole blood samples; however, this may impact test performance.

The lower specificity observed for the CDP RDT, resulting from the false-positive findings, could be explained by cross-reactivity with other pathogens, as reported by the manufacturer. Samples yielding false-negative results exhibited lower ELISA positivity indices for both RDTs, suggesting that the reduced sensitivity may be attributable to low anti–*T. cruzi* antibody titers in this population. Although the observed sensitivities should ideally be further improved, RDTs may still facilitate access to diagnosis for populations that do not reach tertiary-level centers. Indeed, a substantial proportion of positive cases resided in areas of La Matanza located far from the hospital, from which patients were required to travel up to 20 km on at least three occasions (laboratory appointment, blood sample collection, and result retrieval) before receiving specialist care (infectious diseases and/or cardiology).

This study highlights the importance of conducting epidemiological assessments tailored to urban contexts within large healthcare centers. Further population-based studies are needed to expand these findings and improve the management of *T. cruzi* infection. This is particularly relevant in healthcare settings where, in the context of increasing urbanization, most affected individuals now reside. Providing adequate care for individuals with *T. cruzi* infection in large suburban areas of Latin America remains a challenge. It requires interdisciplinary research that addresses the complexity of the disease, rethinks care pathways, and generates evidence on the implementation and evaluation of new tools and technologies developed in recent decades, including rapid diagnostic tests.

## Data Availability

The raw data supporting the conclusions of this article will be made available by the authors, without undue reservation.
